# Quality of life scores using SF-12 and SF-36v2 questionnaires for patients with multidrug-resistant tuberculosis in Vietnam

**DOI:** 10.1186/s12955-026-02479-y

**Published:** 2026-02-06

**Authors:** Nguyen Thi Lien Ha, Nguyen Binh Hoa, Manisha Yapa, Nguyen Thu Anh, Qingbin Li, Vu Hai Dang, Greg J. Fox, Vu Quoc Dat

**Affiliations:** 1 Department of Infectious Diseases, Hanoi Medical University, Hanoi, Vietnam; 2 National Lung Hospital, Hanoi, Vietnam; 3 Faculty of Medicine and Health, The University of Sydney, NSW, Australia; 4 The University of Sydney Viet Nam Institute, Ho Chi Minh city, Vietnam

**Keywords:** Quality of life, Multi-drug resistant tuberculosis, Short-Form 36, Short-Form 12

## Abstract

**Background:**

The health-related quality of life (QoL) assessed by the 12-item Short Form (SF-12) offers a time-efficient alternative to the 36-item version 2 (SF-36 v2). This study aimed to compare the performance of SF-12 and SF-36 v2 among patients with rifampicin-resistant/multidrug-resistant tuberculosis (RR/MDR-TB) in Vietnam.

**Methods:**

A cross-sectional survey was conducted among RR/MDR-TB patients treated in seven provinces between October 2020 and March 2023. Participants completed the SF-36 v2 questionnaire at enrollment. Physical (PCS) and mental component summary (MCS) scores were compared between SF-12 and SF-36 v2. Linear regression assessed the ability of PCS-12 and MCS-12 to predict PCS-36 and MCS-36. Discriminative ability was assessed via receiver operating characteristic (ROC) curves.

**Results:**

This study included 565 participants with a median age of 45.4 years (IQR 44.2–46.5) and a male proportion of 71.7%. 64.1% (362/565) had PCS-36 and 88.3% (499/565) had MCS-36 scores well below and below population norms. The mean scores of PCS-12 and MCS-12 were higher than those of PCS-36 (45.1; 95% CI 44.1–46.1 vs. 43.6; 95% CI 43.1–44.1), and MCS-36 (45.2; 95% CI 43.8–46.6 vs. 34.6; 95% CI 33.9–35.4; *p* < 0.001), respectively. The Intraclass Correlation Coefficients (ICCs) between PCS and MCS scores of SF-12 and SF-36 v2 were 0.6 (95% CI: 0.5–0.6) and 0.5 (95% CI: 0.4–0.5) respectively. The AUC values for comparing the performance of PCS and MCS of two scales were 0.89 and 0.99, respectively. We found the excellent linear correlation between PCS-12 and PCS-36 (*r* = 0.8; *p* < 0.001) and between MCS-12 and MCS-36 scores (*r* = 0.9; *p* < 0.001).

**Conclusion:**

SF-12 had moderate correlation to SF-36 v2 for QoL assessment in RR/MDR-TB patients, though it tends to overestimate QoL in older and male individuals.

**Supplementary Information:**

The online version contains supplementary material available at 10.1186/s12955-026-02479-y.

## Introduction

Tuberculosis (TB) is the leading cause of death among infectious diseases and global targets for TB control are currently significantly behind schedule [1]. In 2023, 8.2 million new TB cases were detected and reported globally, resulting in approximately 1.25 million deaths [1]. The epidemiological burden of these new TB cases are more often attributed to low- and middle-income countries; [2] therefore, countries with significant resource constraints must identify the most effective evidence-based strategies to advance TB control efforts and enhance the quality of treatment for tuberculosis patients. Vietnam has made considerable progress in curbing TB, however, the country is listed by World Health Organization (WHO) as having a high burden for both TB and rifampicin-resistant/multidrug-resistant (RR/MDR-TB) and have faced significant challenges [1].

TB, especially RR/MDR-TB can significantly reduce quality of life (QoL) due to prolonged treatment duration, burden of pills, drug side effects, and requirement of adherence [3, 4]. Additionally, the stigma and the sequalae of tuberculosis often impact patients’ QoL [5]. Improving the QoL has become one of the main goals in the treatment and care of RR/MDR-TB patients [6].

Previous studies in Yemen, South Africa, and Romanni using the SF-36 v2 have shown consistently low health related QoL scores among MDR-TB patients, especially in mental health, influenced by delayed diagnosis and treatment-related adverse events, underscoring the substantial impact of MDR-TB and the need for further evaluation of these measurement tools in this population [3, 7, 8]. In Vietnam, only few small-scale studies assessed QoL among patients with MDR-TB conducted in restricted geographic areas with modest sample sizes [9, 10].

Compared with the original SF-36 v1, SF-36 v2 incorporates several improvements in content and layout, including clearer wording of instructions and selected items and enhances clarity and respondent comprehension0 [11]. Although the SF-36 v2 is a widely accepted instrument for assessing health related QoL, providing standardized measurement and enabling meaningful comparisons across disease populations and with the general population, its length and complexity may pose challenges for specific populations, particularly older adults and individuals with severe health conditions [12].

The shortened SF-12 questionnaire is a 12 question survey that is derived from the SF-36 v2 questionnaire, serves as a substitute for the standard SF-36 v2 questionnaire [13]. The 12 items are derived from the full SF-36 v2 questionnaire and retain coverage of all eight health domains [14]. They are utilized to compute the Physical Component Summary 12 (PCS-12) and Mental Component Summary 12 (MCS-12) scores using a standardized scoring algorithm [15]. The SF-12 questionnaire has been used in both the general population and among patients with chronic diseases [13, 16, 17]. Compared to the SF-36 v2, which takes about 10 min, the SF-12 has the advantage of being able to be completed in less than two minutes [18]. A Vietnamese version of the SF-36 v2 has been validated for assessment of self-reported health status among the Vietnamese [19]. However, given the time constraints of researchers collecting data in a clinical setting, a short questionnaire of SF-12 may provide similar results to the longer questionnaire. This study aimed to compare the SF-12 and SF-36 v2 questionnaires for their correlation in component scores (PCS and MCS) between two scales among RR/MDR-TB patients receiving short term regimens.

## Methods

### Study design

This was a cross sectional study among patients who enrolled in the VSMART trial”. The V-SMART study was a community-based, open-label, parallel-group randomized control trial (RCT) in Vietnam undertaken between January, 2020 and August, 2023 to evaluate the effect of mobile health (mHealth) intervention on MDR-TB treatment outcomes (Trial registration: ACTRN12620000681954 - registered on 15 June 2020) [20]. The trial was conducted at health facilities in the Programmatic Management of Drug-Resistant Tuberculosis (PMDT) Program in seven provinces in Vietnam (An Giang, Can Tho, Da Nang, Hanoi, Ho Chi Minh, Thanh Hoa and Tien Giang provinces). Sites included a mixture of rural and urban populations from across the country, representative of the population of Vietnam.

The target population for this study were patients who met the following inclusion criteria: (1) being 15 years of age and above, (2) having a diagnosis of bacteriologically confirmed pulmonary and/or extrapulmonary rifampicin resistant (RR) or multidrug-resistant (MDR)-TB (defined by positive Xpert MTB/RIF^®^ or phenotypic resistance to both rifampicin and isoniazid in susceptibility testing), and (3) started treatment for MDR-TB with an antibiotoic regimen containing either bedaquiline or amikacin. The treatment decisions were made by the physician at each healthcare facility following the national guidelines for diagnosis and treatment of drug-resistant TB [21]. Patients unable to provide informed consent due to neuropsychiatric disorders were excluded. All participants were invited to complete the SF-36 v2 questionnaire.

### Data collection

Patients completed the Vietnamese SF-36 v2 questionnaire within thirty days of initiation of a standard short-course treatment for MDR-TB, containing either bedaquiline or amikacin - recommended by WHO for eligible patients to improve outcomes and reduce treatment burden [21].

The SF-36 v2 questionnaire included eight domains and was divided into two summary scores, included Physical Component Summary (PCS) with 4 domains of 21 questions (Physical Functioning - PF, Role physical - RP, Bodily pain – BP and General health - GH, ) and Mental Component Summary (MCS) with 4 domains of 14 questions (Vitality - VT, Social Functioning - SF, Role Emotional - RE, and Mental Health - MH) and 1 question of Change in health. ^11^ The questionnaire has been validated in Vietnamese and used to evaluate quality of life in Asian settings [22].

Within 30 days of beginning treatment, the participants were asked to complete the SF-36 v2 themselves. Physicians or research staff who had been trained to conduct the SF-36 v2 questionnaire supported the participants to complete SF-36 v2 questionnaire. To facilitate participation, financial assistance was provided to cover enrollment fees, which included costs associated with completing the required questionnaires. The SF-12 questionnaire is a subset of the SF-36 v2 questionnaire, including 8 domains for 2 summary scores of PSC (PSC-12) and MCS (MCS-12) similar to the SF-36 v2 questionnaire but fewer items for each domain (a total of 12 items) [14]. SF-36 v2 scores was calibrated and transformed to norm-based scores using Smart Measurement^®^ System (upgraded from QualityMetric Health Outcomes™ Scoring Software). SF-12 scores were calculated from items embedded in the SF-36 v2 questionnaire (Appendix 1) and was transformed to a scale from 0 to 100. Therefore, in both SF-12 and SF-36 v2 questionnaires, the total score for each domain ranged between 0 and 100. Higher scores indicate better well-being or better QoL.

The QoL was further categorised into “Well below”, “Below” and “Same or better” indicating a respondent’s norm-based SF score that is 10 points or more below, 5 to less than 10 points and less than 5 points below the age and gender-specific benchmark for Vietnamese general population [23].

### Statistical analysis

Descriptive statistics (mean and standard deviation) were calculated for each of domains of both SF-12 and SF-36 v2 [11, 14]. Known-group validity was assessed by examining patterns of mean PCS and MCS scores across prespecified subgroups expected a priori to differ in HRQoL, including age group, sex, educational level, comorbid diabetes, tuberculosis classification, and treatment history. A supplementary exploratory multivariable analysis using PCS-36 and MCS-36 was also conducted to provide contextual support for these comparisons. We used a linear regression and estimated Pearson’s correlation coefficient between each corresponding domain and its component items of SF-12 and SF-36 v2. The correlation was considered positive and significant when *r* ≥ 0.30 [11]. Paired sample t-test was used to compare the mean scores of PCS-12 and PCS-36 and the mean scores of MCS-12 and MCS-36, respectively. Linear regression was also conducted to evaluate the predictive ability of PCS-12 and MCS-12 for PCS-36 and MCS-36, respectively. PCS-36 and MCS-36 are dependent variables, the corresponding variables of the SF-12 scale are used as independent variables.

Internal consistency of the domains was assessed by the Pearson’s correlation coefficients for the entire scales, each construct, and each factor. The Pearson’s correlation coefficients of 0.9–1 was considered very high correlation, 0.7–0.9 high correlation, 0.5–0.7 moderate correlation, 0.3–0.5 low correlation and 0–0.3 negligible correlation [24]. We hypothesised a priori that PCS-12 and MCS-12 would demonstrate strong positive correlations (Pearson’s *r* ≥ 0.70) with PCS-36 and MCS-36, respectively, reflecting measurement of the same underlying constructs. We further hypothesised at least moderate agreement between SF-12 and SF-36 summary scores, defined as an ICC ≥ 0.50. We used receiver operator characteristic (ROC) curves to evaluate for the discriminative ability of the SF-12 and SF-36 v2 and to assess whether the SF-12 and SF-36 v2 scores could correctly categorize a patient into a severity group of “well below”, “Below” or “Same or better”.

An Area Under the Curve (AUC) indicates perfect discriminatory ability; in which, an AUC of between 0.8 and 1 shows good to excellent ability to discriminate; an AUC of between 0.7 and 0.8 shows fair discriminative ability; an AUC of between 0.60 and 0.70 shows weak ability to discriminate; an AUC below 0.60 indicates a failure to discriminate between groups; and an AUC of 0.50 and suggests the instrument is no more useful to predict the group to which an individual belongs [25]. However, while the adjustment increased AUC, it also caused graphical inconsistencies, suggesting that the improvement was due to the model rather than a real gain in discrimination. This raises concerns that the adjusted AUC may be inflated by statistical adjustments rather than reflecting SF-12’s actual performance. To keep the results clear and meaningful, we reported the unadjusted AUC, which better reflects the predictive relationship between SF-12 and SF-36 v2 without distortions from the model. ICC values were interpreted as poor (< 0.50), moderate (0.50–0.75), good (0.75–0.90), and excellent (> 0.90) agreement [26]. To evaluate the consistency between SF-12 and SF-36 v2, we used Intraclass correlation coefficients (ICC), Pearson correlation coefficient, AUC, and linear regression and its R². While ICC is the most relevant measure for assessing consistency, Pearson’s r helps confirm whether SF-12 and SF-36 v2 are strongly associated, reinforcing that they measure similar constructs. Linear regression and its R² quantify how well SF-12 predicts SF-36 v2 scores, providing additional insight into their alignment. By combining these methods, we ensure a more comprehensive evaluation of how consistently SF-12 aligns with SF-36 v2. The data analysis was performed with the STATA software version 17.0 (Stata Corporation, College Station, TX, USA).

### Ethical issues

The V-SMART trial was granted by the University of Sydney Human Research Ethics Committee (2019/676), the Scientific Committee of the Ministry of Science and Technology, Vietnam (08/QD-HDQL-NAFOSTED), the Institutional Review Board of the National Lung Hospital, Vietnam (13/19/CT-HDDD) and the Institutional Review Board of the Hanoi Medical University, Vietnam (864/GCN-HĐĐĐNCYSH- ĐHYHN). Participants provided written informed consent for their participations.

## Results

A total of 565 Rifampicin resistance/ multidrug-resistant tuberculosis (RR/MDR-TB) patients who completed the SF-36 v2 questionnaire at baseline were included. The median age was 45.4 years (IQR: 44.2–46.5), and 405 (71.7%) were male. Most participants had pulmonary TB (559/565 or 98.9%) and were undergoing their first MDR-TB treatment (342; 64.0%). Comorbidities were reported in 156 patients (27.6%), with diabetes being the most common (124/565 or 21.9%). Demographic characteristics are presented in Table 1.

**Table 1:** The quality of life score based on characteristics of study participants

Characteristic	*n* (%)	PCS-36 score	PCS-12 score	MCS-36 score	MCS-12 score
**Age (years) (** ***n*** ** = 565)**					
18–44	289(51.2)	44.6 ± 11.8	43.0 ± 5.8	45.3 ± 17.4	35.2 ± 9.1
≥ 45	276(48.9)	45.7 ± 12.2	44.3 ± 6.1	45.0 ± 17.4	34.1 ± 8.6
**Sex (** ***n*** ** = 565)**					
Male	405(71.7)	45.1 ± 11.7	43.6 ± 5.9	43.3 ± 17.1	33.9 ± 8.9
Female	160(28.3)	45.2 ± 12.8	43.7 ± 6.2	49.9 ± 17.2	36.7 ± 8.6
**Highest level of education completed (** ***n*** ** = 551)**					
High school and upper	245(44.5)	43.5 ± 11.7	42.8 ± 5.9	45.9 ± 17.8	35.2 ± 8.8
Under High school	306(55.5)	46.4 ± 12.2	44.33 ± 6.0	44.3 ± 17.3	34.1 ± 9.1
**Comorbidity (** ***n*** ** = 565)**					
Diabetes	124(22.0)	46.9 ± 12.0	44.5 ± 5.7	45.7 ± 18.0	34.5 ± 8.6
HIV	16(2.8)	49.2 ± 14.4	45.1 ± 6.9	47.3 ± 12.7	35.4 ± 8.8
Asthma	16(2.8)	447 ± 13.8	45.0 ± 8.1	46.9 ± 14.2	33.9 ± 8.5
**Tuberculosis classification (** ***n*** ** = 565)**					
Pulmonary tuberculosis	559(98.9)	45.1 ± 11.9	43.6 ± 6.0	45.1 ± 17.3	34.6 ± 8.9
Extrapulmonary TB	3(0.5)	54.2 ± 23.7	45.1 ± 8.9	47.9 ± 33.7	38.0 ± 20.2
Pulmonary tuberculosis and extrapulmonary tuberculosis	3(0.5)	38.5 ± 48	41.1 ± 3.9	528 ± 11.0	37.2 ± 4.8
**TB treatment classification (** ***n*** ** = 534)**					
New	342(64.0)	44.8(119)	43.2(5.7)	46.4(17.4)	35.1(8.9)
Relapse or Retreatment after quit treatment	192(36.0)	45.8(12.4)	44.3(6.4)	43.0(16.8)	33.9(8.5)

PCS: Physical Component Summary

MCS: Mental Component Summary

**Fig. 1:**
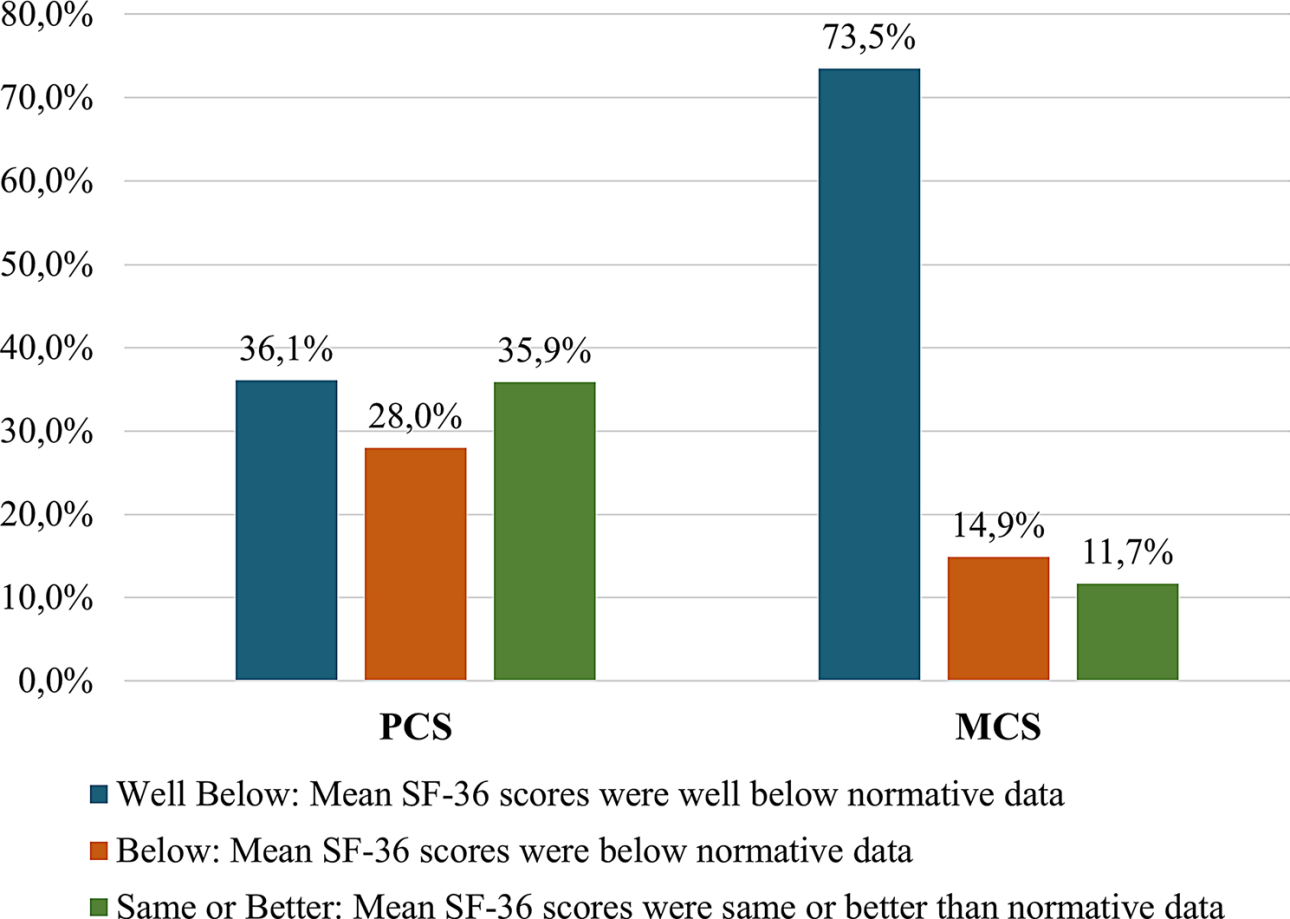
The distribution of SF-36 scores among patients with RR/MDR-TB

Overall, 64.1% and 88.3% of participants had PCS and MCS score, respectively, below Vietnamese population norms (Fig. 1). Mean PCS-12 score (45.1; 95% CI: 44.1–46.1) was significantly higher than PCS-36 score (43.6; 95% CI: 43.1–44.1; *p* < 0.001). Likewise, MCS-12 score (45.2; 95% CI: 43.8–46.6) exceeded MCS-36 score (34.6; 95% CI: 33.9–35.4; *p* < 0.001). Differences in PCS and MCS scores between SF-12 and SF-36 v2 were observed across selected subgroups expected a priori to differ in HRQoL, providing descriptive evidence relevant to known-groups validity (Table 1). Several pre-specified characteristics independently differentiated SF-36 v2 summary scores (Table 2). Descriptive analyses showed differences in SF-36 scores across several participant subgroups (Table 1). However, after adjusting for participant characteristics specified a priori for further assessment of known-group validity, only education and sex remained associated with SF-36 scores. Specifically, educational level is an important determinant of physical health-related quality of life, while sex differences are more prominent in mental health-related quality of life in the study population (Table 2). Other characteristics showed smaller or imprecise associations.

**Table 2:** Factors associated with SF-36 v2 physical and mental component summary scores

Characteristic	PCS-36 β (95% CI)	*p*	MCS-36 β (95% CI)	*p*
Age (≥ 45 years vs. 18–44 years)	0.81 (-0.30–1.93)	0.15	-0.36 **(**-2.04–1.30**)**	0.66
Sex (Female vs. Male)	0.75 (-0.39–1.89)	0.19	**2.55** **(0.84–4.26)**	**< 0.01**
Education (Under high school vs. high school and upper)	**1.21** (**0.15–2.28)**	**0.02**	-0.68 (-2.28–0.90)	0.39
Diabetes (Yes vs. No)	0.57 (-0.67–1.82)	0.36	0.29 (-1.57–2.16)	0.76
Pulmonary TB (Yes vs. No)	0.39 (-4.41–5.20)	0.87	–2.49 (-9.70–4.71)	0.49
New MDR-TB (Yes vs. No)	-0.95 (-1.98–0.07)	0.07	0.81 (-0.72–2.35)	0.29

Values are presented as regression coefficients (β) with 95% confidence intervals

PCS: Physical Component Summary

MCS: Mental Component Summary

These variables were used to adjust intraclass correlation coefficients (ICC) via a two-way mixed effects model. The ICCs between PCS-12 and PCS-36 and between MCS-12 and MCS-36 scores were 0.6 (95% CI: 0.5–0.6) and 0.5 (95% CI: 0.4–0.5) respectively, indicating moderate agreement. SF-12 tended to overestimate QoL in older patients and males, but the correlation between instruments remained moderate (ICC range: 0.5–0.75).

Internal consistency by domain is shown in Table 3. For SF-36 v2, high inter-item correlations were noted in RP (*r* = 0.76–0.86), BP (*r* = 0.81), and RE (*r* = 0.84–0.89). For SF-12, RP (*r* = 0.85) and RE (*r* = 0.86) also demonstrated high internal consistency. Although negative correlations are theoretically possible, all observed correlations were positive. No single domain in either instrument showed moderate or strong correlation with the general health domain.

**Table 3:** Correlation between SF-12 and SF-36 scales in relation to its component items

No	Scale	Range of Pearson’s correlation coefficients with the component items	Pearson’s correlation coefficients with the general health scale
1	Physical functioning		
	SF-12	0.62–0.62	-0.37*
	SF-36	0.10–0.69	0.31*
2	Role-physical		
	SF-12	0.85–0.85	-0.37*
	SF-36	0.76–0.86	0.30*
3	Bodily pain		
	SF-12	-	0.36*
	SF-36	0.81–0.81	-0.36*
4	General health		
	SF-12	-	1.00*
	SF-36	0.23–0.60	1.00*
5	Vitality		
	SF-12	-	-0.17*
	SF-36	0.03–0.69	0.40*
6	Social functioning		
	SF-12	-	-0.18*
	SF-36	0.61–0,61	-0.03
7	Role-emotional		
	SF-12	0.86–0,86	-0.30*
	SF-36	0.84–0.89	0.32*
8	Mental health		
	SF-12	0.32–0,32	-0.16*
	SF-36	0.22–0.66	0.41*

* *p* < 0.05

“-” Pearson’s correlation coefficient was not available since this scale is measured by only one item in the SF-12

**Fig. 2:**
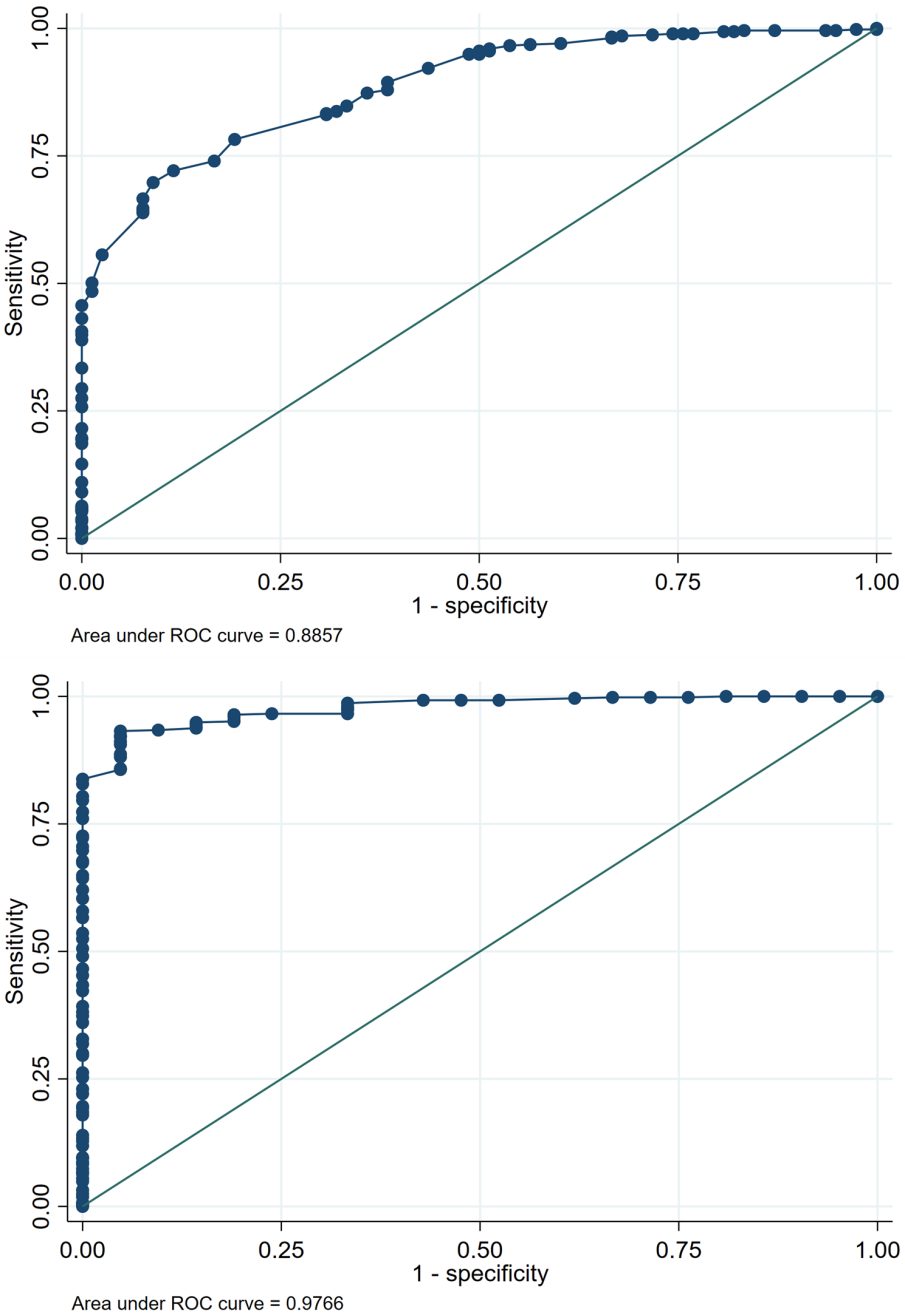
Performance of PCS and MCS components of SF-12 against SF-36 v2 for the measurement of quality of life

**Fig. 3:**
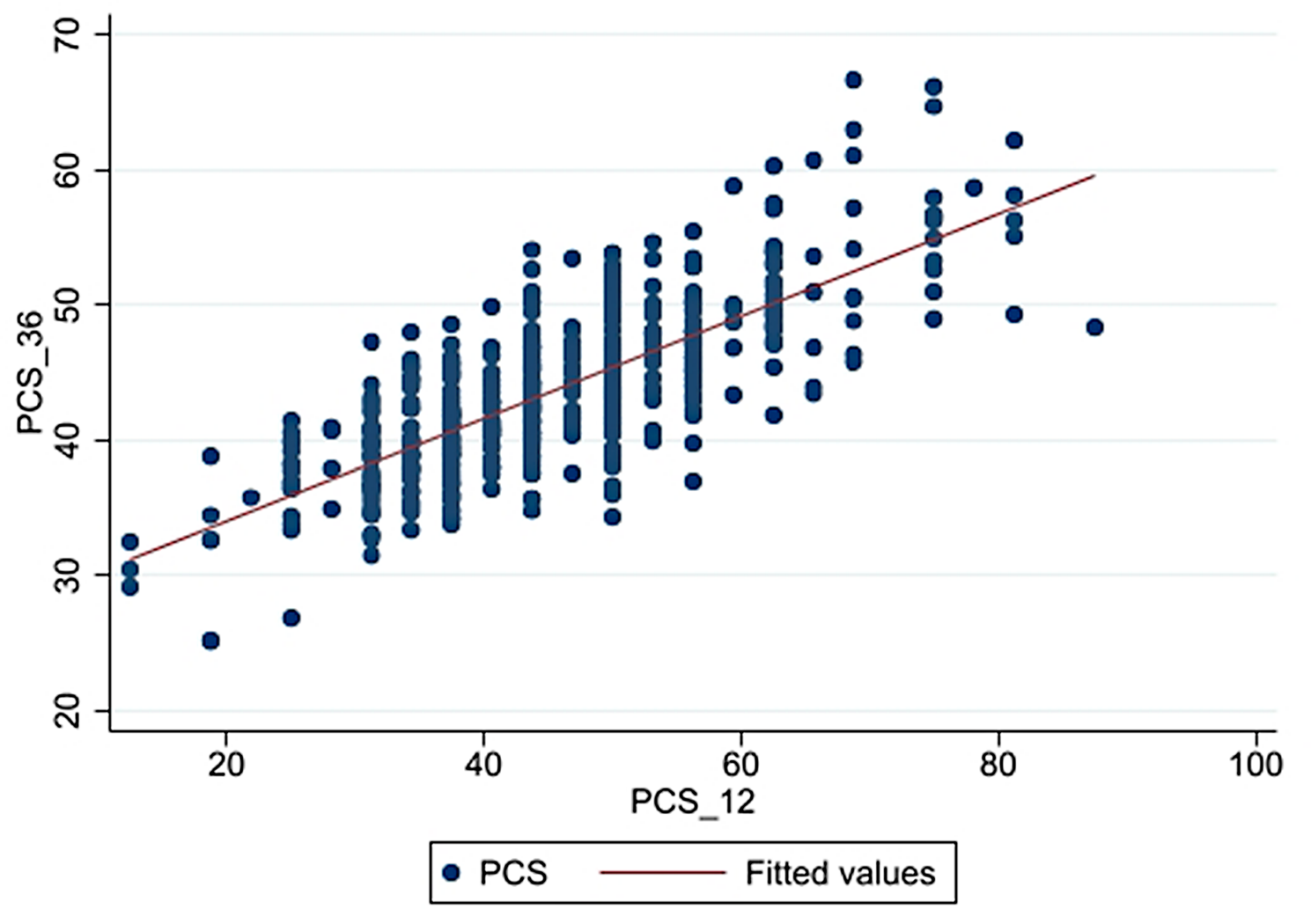
Scatterplot (•) and Pearson correlation (-) between the PCS-12 and the PCS-36 in RR/MDR-TB patients. PCS-12: Physical component summary score of the 12-item Short Form Health Survey. PCS-36: Physical component summary score of the 36-item Short Form Health Survey

**Fig. 4:**
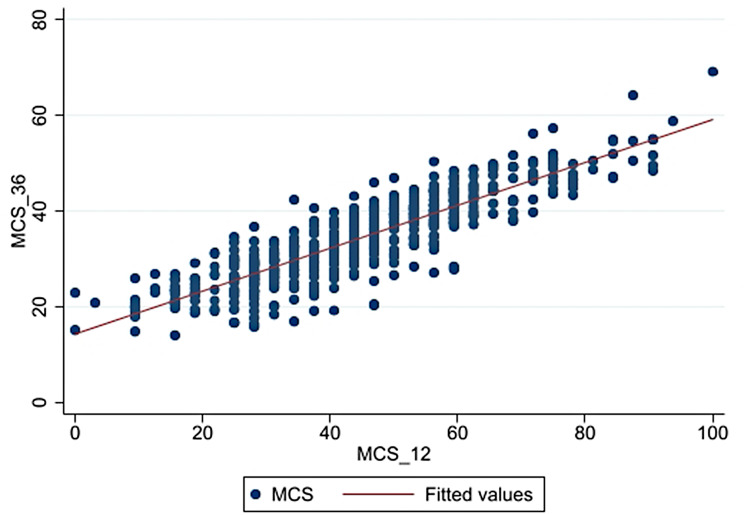
Scatterplot (•) and Pearson correlation (-) between the MCS-12 and the MCS-36 in RR/MDR-TB patients. MCS-12: Mental component summary score of the 12-item Short Form Health Survey. MCS-36: Mental component summary score of the 36-item Short Form Health Survey

Variables with significant differences in PCS and MCS scores between SF-12 and SF-36 v2 were included in ROC analyses. Adjusted analyses confirmed the excellent discriminatory ability of the SF-12, with AUCs of 0.88 for PCS and 0.97 for MCS. For SF-12, sensitivity, specificity, Positive Predictive Value (PPV), and Negative Predictive Value (NPV) were 32.91%, 98.15%, 74.29%, and 90.00% for PCS, and 52.38%, 99.26%, 73.33%, and 98.18% for MCS (Fig. 2). Strong linear correlations were observed between SF-12 and SF-36 v2 scores for both PCS (*r* = 0.76; *p* < 0.001) and MCS (*r* = 0.87; *p* < 0.001) (Figs. 3 and 4).

## Discussion

We found that majority of RR/MDR-TB patients had lower QoL scores than the general population (based on a cut-off of 50 for SF-12 and for SF-36 v2). Factors associated with lower scores included younger age, male, education of under high school level, diabetes, pulmonary TB, and being newly TB diagnosed.

Using SF-36 v2, whilst 36.1% of participants had PCS scores lower than the population norm, 73.5% of participants reported MCS scores below the norm. This highlighted the psychological toll of RR/MDR-TB and the need for early mental health support [27]. This underscores the urgent need for psychological and emotional support interventions as soon as patients are diagnosed with RR/MDR-TB [28].

Correlations between SF-12 and SF-36 v2 component scores varied with strong to moderate correlations (*r* = 0.3–0.9) in RP, BP, RE, SF, and VT domains whilst weak correlations (*r* ≤ 0.4) were observed among SF-12 items and general health in SF-36 v2. Mean scores of PCS and MCS between SF-12 and SF-36 v2 in our study were statistically significant difference however they may not be clinically significant because the minimum clinically important difference for the SF-36 v2 was found to be approximately 5 points for both the PCS and MCS) scores [29]. Some studies compared the components of the SF-12 and SF-36 v2 questionnaires and found no significant difference between the mean summary scores of the physical and mental components, or among scores obtained from the SF-12 when taken alone for an equivalent and independent sample [11, 14].

In our study, ROC analysis showed high predictive accuracy of SF-12 vs. SF-36 v2, with an AUC of 0.9943 (the cut-off of 50 points for Vietnamese general population). PCS-12 and MCS-12 effectively classified patients by level of QoL and explained most variance in PCS-36 and MCS-36 scores. These findings align with previous studies in hypertension, stroke, and dialysis patients, where SF-12 showed high agreement with SF-36 (e.g., ICCs ≥ 0.88), and captured 78–89% of score variance [13, 16, 18].

Various measurements have been proposed to evaluate quality of life, including direct and overall measurement methods utilizing quality of life coefficients related to specific health conditions (such as the time trade-off method, visual analog scale method, and standard gamble method) [30] as well as standardized scales including SF-36, SF-12, EuroQoL-5 dimension-5 level (EQ-5D-5 L), WHO Quality of Life − 100 (WHOQOL-100), and WHO Quality of Life-BREF (WHOQOL-BREF) [31, 32]. Compared to other quality of life instruments, the SF-36 demonstrates strong reliability, good discriminative validity across disease severity and treatment stages, and consistent correlations with both patient- and physician-reported outcomes, making it a robust tool for evaluating health-related quality of life in tuberculosis patients [33]. The SF-36 questionnaire was used to evaluate quality of life across various conditions, including hypertension and dialysis [13, 16].

The SF-36 v2 questionnaire has been validated for evaluating the quality of life of Vietnamese individuals [19, 22]. However, a drawback of this scale is its inclusion of numerous indicators (36 indicators), leading to patient fatigue and distraction during the evaluation process.

Despite of the high burden of TB in Vietnam, the quality of life of tuberculosis patients has not received adequate attention, especially among multidrug-resistant tuberculosis (MDR-TB) patients. Drug resistance in tuberculosis patients has been reported to affect both their physical and mental health [4]. Health-related quality of life improved after the initiation of treatment for MDR-TB, with symptom duration before diagnosis being a predictor of poor quality of life [34]. Given the high prevalence of mental disorders identified in patients with MDR-TB, screening and diagnosing mental health disorders, should be integral parts of MDR-TB care [35].

This study has several important policy implications. Our work aligned with the recent WHO’s recommendations for the collection of adequate data and information to improve understanding of the needs of individuals with TB-associated disabilities to support evidence-based decision-making and to inform the planning and provision of effective, person-centered services [6]. As shown in our study, the low QoL was driven by the low mental health scores; providing psychological support may improve health-related quality of life and cure rates by individual interventions using a patient-centered model and policy-making to implement burden-reducing measures and social support for people with TB.

Our study has some limitations. First, SF-12 questionnaire has not been validated independently in Vietnamese and in this study, the SF-12 scores were calculated from the SF-36 v2 questionnaire rather than interview patients directly with the two questionnaires. Consequently, the advantage of the SF-12 questionnaire’s brevity may not be fully represented. However, the large sample size in our study may yield sufficient power for comparison of the SF-36 v2 and SF-12. Secondly, we can’t provide the analysis of the changes in the QoL overtime, which was difficult for the interpretation of the impact of treatment on the study population and of the participants’ self-assessment of their health with influences of temporary emotions or health concepts.

## Conclusion

The quality of life of Vietnamese individuals with RR/MDR-TB was lower than the population norms. Although the correlation between the SF-12 and SF-36 v2 questionnaires is not perfect, the SF-12 questionnaire proves valuable as an alternative for assessing the quality of life in RR/MDR-TB patients. Its relative simplicity and ease of application make the SF-12 questionnaire suitable for evaluating individuals with a diminished quality of life, including those afflicted with RR/MDR-TB, which is highly beneficial for enhancing tuberculosis treatment effectiveness. However, such assessments necessitate objective evidence.

## Supplementary Information

Below is the link to the electronic supplementary material.

Supplementary Material 1

## Data Availability

The datasets generated and/or analyzed during the current study are not publicly available due to confidentiality agreements but are available from the corresponding author on reasonable request.

## Abbreviations

AUC: Area Under the Curve
BP: Bodily pain
CI: Confidence intervals
EQ-5D-5L: EuroQoL-5 dimension-5 level
GH: General health
HRQoL: Health-related quality of life
ICC: Intraclass correlation coefficient
MCS: Mental Component Summary
MH: Mental health
PCS: Physical Component Summary
PF: Physical functioning
QoL: Quality of life
RR/MDR-TB: Rifampicin resistance/ multidrug-resistant tuberculosis
SF: Social functioning
SF-36: 36-items short form
SF-12: 12-items short form
SF-36 v2: 36-items short form version 2
TB: Tuberculosis
RE: Role emotional
ROC: receiver operator characteristic
RP: Role physical
VT: Vitality
WHO: World Health Organization
