# Supplementary material for: Quality of life scores using SF-12 and SF-36v2 questionnaires for patients with multidrug-resistant tuberculosis in Vietnam
Source: Health Qual Life Outcomes. 2026 Feb 6;24:33. doi: 10.1186/s12955-026-02479-y (PMC12973597; doi:10.1186/s12955-026-02479-y)
Supplement: Supplementary file 1 — Supplementary Material 1 [file 12955_2026_2479_MOESM1_ESM.docx]

**CONSTRUCTION OF THE SF-12**

|  |  | **SF36** | **SF12** |
| --- | --- | --- | --- |
| **PCS** | Physical functioning  PF | Question 3a; Question 3b; Question 3c; Question 3d; Question 3e; Question 3f; Question 3g; Question 3h; Question 3i; Question 3j; | Question 3b;  Question 3d |
|  | Role-physical  RP | Question 4a; Question 4b; Question 4c; Question 4d | Question 4b; Question 4c; |
|  | Bodily pain  BP | Question 7;  Question 8 | Question 8 |
|  | General health  GH | Question 1; Question 11a; Question 11b; Question 11c; Question 11d | Question 1; |
| **MCS** | Vitality  VT | Question 9a; Question 9e; Question 9g;  Question 9i | Question 9e; |
|  | Social functioning  SF | Question 6; Question 10; | Question 10; |
|  | Role-emotional  RE | Question 5a; Question 5b; Question 5c | Question 5b; Question 5c |
|  | Mental health  MH | Question 9b; Question 9c; Question 9d; Question 9f;  Question 9h | Question 9d; Question 9f; |
| **Change in Health** | | Question 2 |  |

The SF-12 is derived from 12 specific items of the SF-36 v2 questionnaire. The corresponding SF-36 items listed above are used to calculate the scores of the SF-12. The numbering and order of the SF-36 questions presented in this table were referenced from Ware, JE, Snow, KK, Kosinski, M., et al. (1993) SF-36 Health Survey Manual and Interpretation Guide. New England Medical Center, the Health Institute, Boston.
